# MIR149 rs2292832 and MIR499 rs3746444 Genetic Variants Associated with the Risk of Rheumatoid Arthritis

**DOI:** 10.3390/genes14020431

**Published:** 2023-02-08

**Authors:** Yasir Ali, Yangchao Chen, Zia Ul Islam, Aisha Aman, Mashal M. Almutairi, Abdulaziz Alouffi, Aymen Mohammed, Aftab Ali Shah, Zia Ur Rehman, Ibrar Hussain, Abid Ali, Fazal Jalil

**Affiliations:** 1 Department of Biotechnology, Abdul Wali Khan University Mardan, Mardan 23200, Pakistan; 2 School of Biomedical Sciences, Chinese University of Hong Kong, Hong Kong 999077, China; 3 Department of Pharmacology and Toxicology, College of Pharmacy, King Saud University, Riyadh 11451, Saudi Arabia; 4 King Abdulaziz City for Science and Technology, Riyadh 12354, Saudi Arabia; 5 Division of Molecular Therapeutics and Formulation, School of Pharmacy, University of Nottingham, Nottingham NG7 2RD, UK; 6 Department of Biotechnology, Faculty of Biological Sciences, University of Malakand, Chakdara 18800, Pakistan; 7 Peshawar Medical College, Peshawar 25160, Pakistan; 8 Department of Medicine, Lady Reading Hospital (LRH), Peshawar 25000, Pakistan; 9 Department of Zoology, Abdul Wali Khan University Mardan, Mardan 23200, Pakistan

**Keywords:** rheumatoid arthritis, miRNAs, variants, genotyping

## Abstract

Introduction: MicroRNAs (miRNAs) are small non-coding RNAs that play a key role in post-transcriptional modulation of individual genes’ expression. Several miRNA variants from different populations are known to be associated with an increased risk of rheumatoid arthritis (RA). Aim: This study was undertaken with the aim to investigate the association of single nucleotide variants; namely, rs2292832, rs3746444, rs11614913, rs1044165, and rs767649 of MIR149, MIR499, MIR196, MIR223, and MIR155, respectively, with RA in the Pakistani population. Methods: A case-control study was performed by recruiting and genotyping a total of 600 individuals (300 cases and 300 controls) for these five variants using a TaqMan single-nucleotide polymorphism (SNP) genotyping assay. The resultant genotypic data was statistically analyzed through a chi-squared test for its association with RA under different inheritance models. Results: We found a significant association of rs2292832 with RA at genotypic (co-dominant (*p* < 0.0001), dominant (CC vs. TT + CT: OR 2.063 (1.437–2.962); *p* = 0.0001), recessive (TT vs. CT + CC: OR 0.376 (0.259–0.548); *p* < 0.0001)), and allelic (allele C) levels ((OR 0.506 (0.402–0637); *p* < 0.0001)). Similarly, the rs3746444 showed a significant association with RA under co-dominant (*p* = 0.0001), dominant (GG vs. AA + AG: OR 5.246 (3.414–8.061); *p* < 0.0001), recessive (AA vs. GG + AG: OR 0.653 (0.466–0.916); *p* = 0.014), and additive models (G vs. A; OR 0.779 (0.620–0.978); *p* = 0.03). However, we did not observe any significant association of rs11614913, rs1044165, or rs767649 with RA in our subjects. Conclusion: To our knowledge, this was the first study that investigated and found an association between functional polymorphisms in miRNAs and RA in the Pakistani population.

## 1. Introduction

Rheumatoid arthritis (RA) is a chronic systemic autoimmune disease that affects 0.5–1% of the world population with regional variation. The rate of occurrence is three times higher in women than in men [1]. Because it is a chronic immune-mediated inflammatory disease, it results in progressive joint damage, cartilage destruction, and bone and synovial hyperplasia—ultimately leading to extra-articular manifestation and multiple co-morbidities [2,3]. The etiology of RA is complex, and several genetic, environmental, hormonal, infectious, and lifestyle factors that contribute to the pathogenies of RA have been identified [4]. The dynamic interactions between the environment, genotype, and phenotype are likely to be mediated by the epigenetic process [5]. Accumulating manifestations have suggested that aberrant epigenetic alterations play a significant role in the dysregulation of T cells and hence contribute to the emergence of RA [6]. Over the last few decades, more than 150 SNPs present in over 70 genes/loci have been reported for RA through genome-wide association studies (GWAS) and candidate-gene approaches [7,8,9,10,11]. Some of these variants were found in microRNAs (miRNAs), which are an important class of epigenetic factors involved in many autoimmune diseases that include RA [12,13].

MicroRNAs, which are approximately 20–22 nucleotides long, are a class of small non-coding RNAs involved in the post-transcriptional regulation of gene expression [14] in diverse cellular and metabolic pathways; they exert their effects by binding to and silencing their target mRNA [15,16]. The single nucleotide changes in miRNA sequences might change miRNA–mRNA target interaction, thereby deregulating or dysregulating the expression of target genes [17]. Due to their key roles in regulating various biological processes such as cell proliferation, apoptosis, metabolism, stress resistance, the cell cycle, chromosome architecture, stem cell maintenance, etc., their aberrant expression and function have been implicated in a variety of human diseases that include cancer and autoimmune disorders [18,19]. The deregulation of miRNAs results in the abnormal behavior of a variety of cells (T lymphocytes and peripheral blood mononuclear resident cells), which causes inflammation that leads to the degradation of the extracellular matrix (ECM) and damage to synovial fibroblasts and osteoclasts (key effector cells of joint destruction in RA) [12,20]. However, several studies have determined—with conflicting results—the correlation between miRNA variants (rs2292832, rs3746444, rs11614913, and rs767649) and the risk of rheumatoid arthritis [21,22,23,24,25,26,27,28]. Upon successful establishment of their roles, miRNAs can serve as potential therapeutic targets for these conditions [12,13]

For successful treatment, early diagnosis plays an important role, especially in patients with risk of poor prognosis (such as high disease activity, the presence of autoantibodies, and early joint damage) [29]. Although it is now possible to diagnose RA using standard diagnostic criteria, for many the early diagnosis is still undifferentiated arthritis at the time of first presentation in the absence of suitable biomarkers—often, this is the most challenging to find [30]. Accumulating evidence of the role of miRNAs in RA and their aberrant expression in the inflamed synovium and circulation of such patients [31] holds promise for their potential use as novel molecular diagnostic markers for RA [32].

Given the role of miRNAs in autoimmune diseases, particularly RA, we investigated miRNA variants (rs2292832, rs3746444, rs11614913, rs1044165, and rs767649 of MIR149, MIR499, MIR196, MIR223, and MIR155, respectively) for their association with the risk of RA in the Pakistani population.

## 2. Materials and Methods

### 2.1. Ethical Approval of the Study

Before enrolling in the study, informed consent was obtained from the patients and controls. Approval for the study was obtained from the ethical committee of Abdul Wali Khan University Mardan (AWKUM) and Lady Reading Hospital (LRH), Peshawar, Pakistan.

### 2.2. Study Population

The study included 600 individuals: 300 cases (mean age 41.3 ± 13.75) and 300 controls (mean age 41.6 ± 12.3 years). Samples of RA cases were recruited from LRH, Peshawar, Pakistan. RA was diagnosed according to the criteria of the ACR/European League Against Rheumatism (EULAR) 2010 [33]. The control group consisted of healthy, age-matched subjects with no history of autoimmunological diseases. The clinical characteristics of RA and controls are summarized in Table 1.

### 2.3. Genomic DNA Extraction and Genotyping of Selected SNPs Using TaqMan Assay

Venous blood samples were collected from all of the study subjects via venipuncture. Genomic DNA was isolated from the peripheral blood of controls and patients using an organic phenol–chloroform procedure, and the samples were stored at −80° for future analysis [34]. The rs2292832 in MIR149, rs3746444 in MIR499, rs11614913 in MIR196, rs1044165 in MIR223, and rs767649 in MIR155 were genotyped using a TaqMan SNP genotyping assay. DNA amplification was performed in 384-well plates using an ABI Q6 system (Applied Biosystems, Waltham, MA, USA).

### 2.4. Statistical Analysis

A chi-squared test was used to determine the Hardy–Weinberg equilibrium (HWE) status of the obtained SNP genotypes. The association between the SNPs and the risk of RA was evaluated using the odds ratio (OR) at a 95% confidence interval (95%CI). Genotype and allele frequencies were determined using direct counting and compared between patients and controls by using chi-squared and Fisher’s exact tests. All co-dominant, homozygous dominant, homozygous recessive, heterozygous, and additive models were used to assess the risk of RA. The genotypes were compared to obtain the *p*-value for all three SNP genotypes. A *p*-value of 0.05 was set as the statistical significance.

### 2.5. In Silico Analysis of miRNAs’ Primary Structures

**Figure 1:**
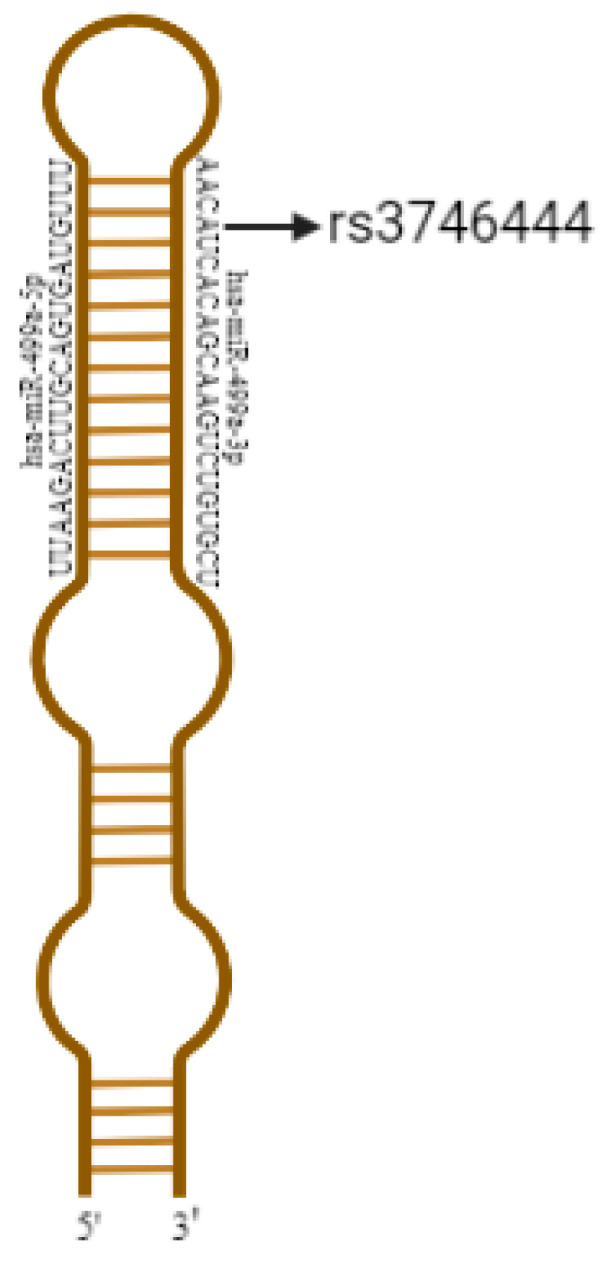
The structure of MIR499 and the maturity of sequence has-mir-499-3p representing all SNPs.

The following list provides information regarding the screened MIR149 rs2292832, MIR499 rs3746444, and MIR196 rs11614913, as well as their gene names, mature miRNAs, respective chromosome positions, coded alleles, other alleles, and minor allele frequencies, can be found in Table 2. Figure 1 illustrate the positions of the seed region and rs3746444 in the hsa-mir-499-3p. A study was conducted to determine whether the rs2292832, rs3746444, and rs11614913 variants affected the primary structure of MIR149, MIR499, and MIR196, respectively. The RNAeval web server was used to calculate the energies of the RNA sequences. The Vienna RNA website was used to examine RNA secondary and centroid structures.

## 3. Results

### 3.1. Genetics Analysis

The genotype distribution of miRNA variants with an RA risk was determined using co-dominant, homozygous dominant, homozygous recessive, heterozygous, and additive models (see Table 3).

The genotype distribution for SNP rs2292832 in MIR149 was significant with RA in the co-dominant (*χ*^2^ = 31.23; *p* < 0.0001), homozygous dominant (CC vs. TT + CT; OR 2.063 (1.437–2.962); *p* = 0.0001), and homozygous recessive (TT vs. CT + CC; OR 0.376 (0.259–0.548); *p* < 0.0001) models, as well as in the distribution of allele C in the additive model (C vs. T; OR 0.506 (0.402–0637); *p*< 0.0001). Similarly, the genotype distribution for SNP rs3746444 in MIR499 showed a significant association with RA in the co-dominant (*χ*^2^ = 59.19; *p* = 0.0001), homozygous dominant (GG vs. AA + AG; OR 5.246 (3.414–8.061); *p* < 0.0001), homozygous recessive (AA vs. GG + AG; OR 0.653 (0.466–0.916); *p* = 0.014), and additive models (G vs. A; OR 0.779 (0.620–0.978); *p* = 0.03). The genotypic distribution of “rs11614913” in MIR196 was more or less random across cases and controls and showed no significant association with RA in the co-dominant (*χ*^2^ = 2.35; *p* = 0.30), homozygous dominant (CC vs. TT + CT; OR 0.930 (0.667–1.298); *p* = 0.73), homozygous recessive (TT vs. CC + CT; OR 0.826 (0.563–1.213); *p* = 0.38), and additive models (C vs. T; OR 1.049 (0.834–1.318); *p* = 0.72). This was also the case for “rs1044165” in MIR223, which showed no meaningful association in the co-dominant model (**χ**^2^ = 2.36; *p* = 0.30), homozygous dominant model (GG vs. AA + AG; OR 1.116 (0656–1.896); *p* = 0.78), homozygous recessive model (OR 0.368 (0.096–1.404); *p* = 0.22), or additive model (A vs. T; OR 0.788 (0.488–1.274); *p* = 0.39). In the same way, no significant differences in the distribution of the genotype for rs767649 in MIR155 across the cases and controls were observed in the co-dominant (χ^2^ = 4.11; *p* = 0.12), homozygous dominant (TT vs. CC + CT; OR 1.319 (0.925–1.88); *p* = 0.14), homozygous recessive (CC vs. TT + CT; OR 2.02 (0.925–8.157); *p* = 0.50), or additive models (C vs. T; OR 0.833 (0.607–1.144); *p* = 0.29).

### 3.2. In Silco Analysis of miRNAs

The rs374644 variant was located in the seed region AC (A/G) UCAC at the position of 20;34,990,448 in the mature sequence of has-mi-499a-3p. The miRNA loop structure increased by one base pair due to this change. The thermodynamic Structure Prediction tool was used to compare the normal and mutant primary miRNA structures, and alterations were observed for the parameters being studied, as shown in Table 4. In the primary structure of miRNA, the rs11614913 variant in MIR196A2 resulted in the substitution of cytosine for uracil. The effect of rs2292832 on the secondary structure of pre-miR-149 **was** studied using the RNAWeb Server. The precursor miR-149 with and without the rs2292832 variant was modeled to determine the minimum free energy (MFE) and centroid structures. Based on the different alleles of rs2292832, no structural modification was observed for MIR149.

## 4. Discussion

Rheumatoid arthritis (RA) is characterized as a vital autoimmune disease that hampers the normal physical activities of patients [35,36]. RA causes inflammation in joints, which results in damage to the articular cartilage along with synovial hyperplasia, ultimately leading to consistent pain and permanent disability. The heritability of RA has been reported to be about 60%, whereas the remaining 40% could be due to environmental and other factors [37]. Genome-wide association studies (GWAS) are a common method for elucidating the underlying genetic risk factors. The major histocompatibility complex (MHC) and non-MHC loci have been identified to account for ~23% of the genetic risk factors for RA, which indicates that there are still undiscovered genetic risk factors [7]. Association studies have reported >150 SNPs located at more than 70 gene loci, which provides a glimpse of the underlying genetic factors [7,8,38,39].

The exact etiology and pathogenesis of RA, which are highly complex and involve genetic susceptibility, environmental factors, and epigenetic changes, are still incomplete. miRNAs play a crucial role in maintaining the normal functions of the immune system. In recent decades, research has revealed that dysregulated miRNAs play a role in autoimmune disease pathogenesis because they regulate a wide range of cellular functions at the post-transcriptional level [40]. Recent studies revealed that the occurrence of autoimmune illnesses, particularly the emergence of RA, may be influenced by epigenetic processes such as histone changes, DNA methylation, and microRNAs [41]. Small non-coding miRNAs are the important components of complex genetic regulatory networks. The SNPs or mutations that occur in the miRNA gene region can affect the properties of miRNAs and lead to a hereditary defect in the protein’s translation of a target mRNA [42].

In this study, a population-based association of rs2292832, rs3746444, rs11614913, rs1044165, and rs767649 genotypes with RA was investigated in the Pakistani population. The SNPs rs2292832 in MIR149 and rs3746444 in MIR499 were found to be significantly associated with RA in this population. The gene for MIR149 was located at 2q37.3 and contains one exon which code MIR149 and showed polymorphisms. The pre-miRNA SNP rs2292832 in MIR149 is thought to contribute to RA pathogenesis. The current study found a significant (*p* < 0.0001) association of rs2292832 in MIR149 with RA in the Pakistani population. Similarly, a case-control study (200 RA patients and 120 controls) that was conducted in the eastern Chinese Han population by Xiao et al. using the RFLP-PCR method reported a significant association with RA at the genotypic and allelic levels [21]. Table 5 shows a list of manuscripts referred in this study. 

The rs4746444 in MIR499 is located within the mature miRNA region, thereby altering the pre-miRNA maturation process and binding sites for target mRNA [43]. The MIR499 polymorphism rs3746444 was reported to be involved in RA risk. Several studies have shown that HLADRB1 alleles that encode shared epitope (SE) and STAT4 genes were significantly associated with RA. A target of MIR499 called regulatory factor X4 (RFX4) can influence HLADRB1 expression. Interestingly, we reported in a previous study that HLADRB1 rs6660895 and STAT4 rs7574865 were strongly associated with RA in the Pakistani population (Jalil et al. 2013). Another target was the *PADIi4* gene, which encodes the PADI4 enzyme. The synovial tissue and hematological cells of RA patients contained PADI4 mRNA, and it was highly overexpressed in the blood of RA patients. Additionally, the PADI4 enzyme generates autoantigens, which are citrullinated peptides that are specifically recognized by anti-CCP [44]. Inhibition of citrullination occurs when arginine is deaminated to citrulline in the presence of calcium ions [45]. As a result, MIR499 might play a significant role in the pathogenesis and severity of RA.

Previously, the association of rs3746444 in the MIR499 study was conducted in several populations and demonstrated a positive association with RA. For the first time, MIR499 rs3746444 was reported by Yang et al. to be associated with RA in the Chinese Han population [22]. Another case-control study that was conducted by Hashemi et al. found a significant association with RA in the Iranian population [23]. Amal et al. showed a significant association of rs3746444 in MIR499 with RA in Egyptian females [24], and Fatah et al. showed a positive association with RA in the Egyptian population [25]. Ayeldeen et al. also found a significant association with RA in the Egyptian population [46]. Recently, rs3746444 in MIR499 was genotyped in 550 RA patients and 550 controls of Pakistani origin; the study reported a significant association [47]. Rs3746444 in MIR499 was significantly associated with the genotypic (*p* = 0.0001, *p* < 0.05) and allelic levels with RA in this study. All these studies were in close agreement with the previous ones that established the association of rs3746444 in MIR499 with RA.

MIR196a2 is located on chromosome 12q13.13 and is expressed from an intergenic region in *HOX* gene clusters. Rs11614913 is located at the passenger strand near the processing site at nucleotide 78. Only the expression of MIR196a2 has been deregulated in RA synovial fibroblasts (RASFs) with no previous association study of its variants with RA susceptibility [48]. The association of rs11614913 in an MIR196a2 study was conducted for the first time in the Egyptian population by Toraih et al.; the researchers found an insignificant association with RA susceptibility [26]. Another extended study was conducted by Aleman-Avila et al. that found no association of rs11614913 in MIR196a2 in the Mexican population [27]. The association of rs11614913 in MIR-196a2 in the Pakistani population was found to be insignificant, and our results conformed to the previous results of Toraih et al., 2016 and Aleman-Avila et al., 2017.

The rs767649 in the pre-MIR155 promoter region, which affects nuclear factor-kappa B (NF-kB) binding as well as several regulatory factors attached to MIR155, has been shown to be involved in RA pathogenesis [49]. The association of rs7676649 in MIR155 in the Egyptian population was reported by Shaker et al., who established a positive association between RA patients and controls at the genotypic and allelic levels [28]. In this study, we did not find a positive association of rs7676649 in MIR155 between the RA and control samples from in the Pakistani population.

The current study had some limitations. This was a single-cohort-based study; thus, the findings need to be validated in a larger extended cohort with demographic characteristics. Additionally, the current study focused only on seropositive RA, therefore no demographic data were collected. To confirm these findings, the results should be independently replicated in populations with demographic data.

In the nutshell, this study genotyped the miRNA SNPs—including rs2292832, rs3746444, rs11614913, rs1044165, and rs767649—in Pakistani RA patients and in healthy controls to determine their association with RA and to aid in determining the genetic background of RA in the Pakistani population. The study revealed that two SNPs (rs2292832 in MIR149 and rs3746444 in MIR499) are likely to have a role in the pathogenesis of RA in the Pakistani population.

## Figures and Tables

**Table 1:** Characteristics of Rheumatoid Arthritis Patients and Controls.

Variable	Cases (*n* = 300)	Controls (*n* = 300)	*p*-Value
Gender (male/female)	113/187	119/181	0.6121
Mean age in years (±SD)	43.5 (±14.5)	42 (±12.6)	0.217
Mean disease duration in years (±SD)	4.1 (±3.7)	-	-
Sero-positive antibody (Me)	100% (RF-positive)	-	-
Mean ESR (±SD)	40.60 (±15.8)	-	-

**Table 2:** Detailed information regarding the miRNAs.

SNP ID	miRNA	Names of Mature miRNA Sequences	Chromosome No.	miRNA Location (Coordinates)	Coded Allele	Other Allele	MAF
rs2292832	MIR149	hsa-miR-149-5p	2	240456001-240456089	T	C	0.38
		hsa-miR-149-3p					
rs374644	MIR499A	hsa-miR-499-5p	20	34990376-34990497 [+]	A	G	0.12
		hsa-miR-499-3p					
rs11614913	MIR196a2	hsa-miR-196a-5p	12	53991738-53991847 [+]	C	T	0.49
		hsa-miR-196a-3p					
rs1044165	MIR223	hsa-miR-223-5p	X	66018870-66018979	G	A	0.00
		hsa-miR-223-3p					
rs767649	MIR155	hsa-miR-155-5p	21	25573980-255740444	T	C	0.29

**Table 3:** Association of RA risk-associated miRNAs in Pakistani case-control study.

miRNA SNPs	Models	Genotypes	Cases (300)	Controls (300)	Odds Ratio	*χ*^2^-Value	*p*-Value
MIR149 rs2292832	Co-dominant	TT CT CC	55 (18.4%) 136 (45.4%) 109 (36.3%)	112 (37.4%) 123 (41.0%) 65 (21.6%)	-	31.23	<0.0001
	Dominant	CC TT + CT	109 (36.3%) 291 (63.7%)	65 (21.6%) 235 (78.4%)	2.063 1.437–2.962	-	0.0001
	Recessive	TT CC + CT	55 (18.4%) 245 (81.6%)	112 (37.4%) 188 (62.6%)	0.376 0.259–0.548	-	<0.0001
	Heterozygous	CT CC + TT	136 (45.4%) 164 (54.6%)	123 (41.0%) 177 (59%)	1.267 0.919–1.748	-	0.16
	Additive	C T	264 (44.0%) 354 (56.0%)	347 (57.8%) 253 (42.2%)	0.506 0.402–0.637	-	<0.0001
MIR499 rs3746444	Co-dominant	AA AG GG	72 (24.0%) 110 (36.7%) 118 (39.3%)	129 (43.0%) 138 (46.0%) 33 (11.0%)	-	59.19	0.0001
	Dominant	GG AA + AG	118 (39.3%) 182 (60.7%)	33 (11.0%) 267 (89.0%)	5.246 3.414–8.061	-	<0.0001
	Recessive	AA GG + AG	72 (24.0%) 228 (76.0%)	129 (43.0%) 171 (57.0%)	0.653 0.466–0.916	-	0.014
	Heterozygous	AG AA + GG	110 (36.7%) 190 (62.3%)	138 (46.0%) 162 (54.0%)	0.679 0.490–0.942	-	0.025
	Additive	A G	254 (42.4%) 346 (57.6%)	291 (58.5%) 309 41.5%)	0.779 0.620–0.978	-	0.03
MIR196a2 rs11614913	Co-dominant	CC CT TT	107 (35.7%) 132 (44.0%) 61 (20.3%)	112 (37.3%) 115 (38.3%) 73 (24.4%)	-	2.35	0.30
	Dominant	CC TT + CT	107 (35.7%) 193 (64.3%)	112 (37.3%) 188 (42.7%)	0.930 0.667–1.298	-	0.73
	Recessive	TT CC + CT	61 (20.3%) 239 (79.6%)	73 (24.4%) 227 (75.6%)	0.826 0.563–1.213	-	0.38
	Heterozygous	CT CC + TT	132 (44.0%) 168 (56%)	115 (38.3%) 185 (61.7%)	1.264 0.912–1.751	-	0.18
	Additive	C T	346 (57.7%) 254 (42.3%)	339 (56.5%) 261 (43.5%)	1.049 0.834–1.318	-	0.72
MIR223 rs1044165	Co-dominant	AA AG GG	3 (1.0%) 26 (8.7%) 271 (90.3%)	8 (2.7%) 24 (8.0%) 268 (89.3%)	-	2.369	0.30
	Dominant	GG AA + AG	271 (90.3%) 29 (9.7%)	268 (89.3%) 32 (10.7%)	1.116 0.656–1.896	-	0.78
	Recessive	AA GG + AG	3 (1.0%) 297 (99.0%)	8 (2.7%) 292 (97.3%)	0.368 0.096–1.404	-	0.22
	Heterozygous	AG AA + GG	26 (8.7%) 274 (91.3%)	24 (8.0%) 276 (92.0%)	1.091 0.611–1.948	-	0.88
	Additive	A G	32 (5.3%) 568 (94.7%)	40 (6.7%) 560 (93.3%)	0.788 0.488–1.274	-	0.39
MIR155 rs767649	Co-dominant	CC CT TT	6 (2.0%) 72 (24.0%) 222 (74.0%)	3 (1.0%) 92(30.7%) 205 (68.3%)	-	4.116	0.12
	Dominant	TT CC + CT	222 (74.0%) 78 (26.0%)	205 (68.3%) 95 (31.7%)	1.319 0.925–1.88		0.14
	Recessive	CC TT + CT	6 (2.0%) 294 (98.0%)	3 (1.0%) 297 (99.0%)	2.02 0.50–8.157	-	0.50
	Heterozygous	CT CC + TT	72 (24.0%) 228 (76%)	92 (30.7%) 208 (69.3%)	0.714 0.497–1.025	-	0.08
	Additive	C T	84 (14.0%) 516 (86.0%)	92 (16.3%) 502 (83.7%)	0.833 0.607–1.144	-	0.29

**Table 4:** Thermodynamic free energy differences between reference mutated sequences.

Parameters	MIR149 rs2292832		MIR499A rs3746444		MIR196A2 rs11614913	
Sequences	Reference Sequence	Mutated Sequence	Reference Sequence	Mutated Sequence	Reference Sequence	Mutated Sequence
Ensemble thermodynamic free energy	−54.29 kcal/mol	−56.49 kcal/mol	−64.92 kcal/mol	−59.55 kcal/mol	−52.02 kcal/mol	−42.82 kcal/mol
Ensemble diversity	7.21	7.20	9.65	13.42	7.18	4.86
Optimal secondary structure with the lowest free energy	−52.70 kcal/mol	−54.90 kcal/mol	−63.20 kcal/mol	−57.70 kcal/mol	−49.90 kcal/mol	−41.60 kcal/mol
Secondary structure of the centroid	−52.70 kcal/mol	54.90 kcal/mol	−63.00 kcal/mol	−54.80 kcal/mol	−41.30 kcal/mol	−44.30 kcal/mol
Ensemble MFE structure frequency	7.56%	7.57%	6.14%	4.96%	6.14%	13.85%

**Table 5:** The basic characteristics of the retrieved studies.

Author	Year	Country	Disease	Control Source	Genotype Method	Cases	Controls	HWE
MIR149 rs22928323								
Xiao	2015	China	RA	HB	PCR-RFLP	200	120	No
MIR499 rs3743444								
Yang	2012	China	RA	PB	PCR-RFLP	208	240	Yes
Hashemi	2013	Iran	RA	PB	T-ARMS-PCR	104	110	Yes
Amal	2013	Egypt	RA	PB	PCR-RFLP	217	245	Yes
Zhang	2013	China	RA	HB	MALDI-TOP MS	206	466	Yes
Yang	2016	China	RA	PB	TaqMan	386	576	Yes
Toraih	2016	Egypt	RA	PB	Real-time PCR	95	200	No
Aleman-avila	2017	Mexico	RA	PB	TaqMan	412	486	Yes
Ayeldeen	2018	Egypt	RA	PB	Real-time PCR	52	56	Yes
Fataah	2018	Egypt	RA	PB	PCR-RFLP	100	100	Yes
MIR196a rs 11614913								
Toraih	2016	Egypt	RA	PB	Real-time PCR	95	200	Yes
Aleman-avila	2017	Mexico	RA	PB	TaqMan	412	486	No
MIR155 rs7676649								
Shaker	2019	Egypt	RA	PB	TaqMan	79	78	Yes

## Data Availability

All data generated or analyzed during this study were included in this article. Further inquiries can be directed to the corresponding author.
